# Phage Cocktail Targeting STEC O157:H7 Has Comparable Efficacy and Superior Recovery Compared with Enrofloxacin in an Enteric Murine Model

**DOI:** 10.1128/spectrum.00232-22

**Published:** 2022-05-10

**Authors:** Yuxin Wang, Dinesh Subedi, Jin Li, Jiaoling Wu, Jianluan Ren, Feng Xue, Jianjun Dai, Jeremy J. Barr, Fang Tang

**Affiliations:** a MOE Joint International Research Laboratory of Animal Health and Food Safety; Key Laboratory of Animal Bacteriology, Ministry of Agriculture; and College of Veterinary Medicine, Nanjing Agricultural University, Nanjing, China; b School of Biological Sciences, Monash University, Victoria, Australia; c School of Life Science and Technology, China Pharmaceutical University, Nanjing, China; University of Tennessee

**Keywords:** phage cocktail, antibiotics, gut microbiota, therapy

## Abstract

O157:H7 is the most important Shiga toxin-producing Escherichia coli (STEC) serotype in relation to public health. Given that antibiotics may contribute to the exacerbation of STEC-related disease and an increased frequency of antibiotic-resistant strains, bacteriophage (phage) therapy is considered a promising alternative. However, phage therapy targeting enteric pathogens is still underdeveloped with many confounding effects from the microbiota. Here we comprehensively compared the therapeutic efficacy of a phage cocktail with the antibiotic enrofloxacin in a mouse model of STEC O157:H7 EDL933 infection. Enrofloxacin treatment provided 100% survival and the phage cocktail treatment provided 90% survival. However, in terms of mouse recovery, the phage cocktail outperformed enrofloxacin in all measured outcomes. Compared with enrofloxacin treatment, phage treatment led to a faster elimination of enteric pathogens, decreased expression levels of inflammatory markers, increased weight gain, maintenance of a stable relative organ weight, and improved homeostasis of the gut microbiota. These results provide support for the potential of phage therapy to combat enteric pathogens and suggest that phage treatment leads to enhanced recovery of infected mice compared with antibiotics.

**IMPORTANCE** With the increasing severity of antibiotic resistance and other adverse consequences, animal experiments and clinical trials investigating the use of phages for the control and prevention of enteric bacterial infections are growing. However, the effects of phages and antibiotics on organisms when treating intestinal infections have not been precisely studied. Here, we comprehensively compared the therapeutic efficacy of a phage cocktail to the antibiotic enrofloxacin in a mouse model of STEC O157:H7 EDL933 infection. We found that, despite a slightly lower protection rate, phage treatment contributed to a faster recovery of infected mice compared with enrofloxacin. These results highlight the potential benefits of phage therapy to combat enteric infections.

## INTRODUCTION

Shiga toxin-producing Escherichia coli (STEC) O157:H7 is a common zoonotic pathogen, which can cause hemorrhagic colitis (HC) and hemolytic uremic syndrome (HUS) in humans, with more severe cases complicated by renal failure and subsequent death (1). STEC O157:H7 is primarily found as part of the gut microbiota of cattle and is transmitted to humans through contaminated food, water, and direct contact with infected people or animals (2). It was reported that STEC isolates from animals and meat in China showed multidrug resistance (3). Vidovic et al. (4) reported that 2% and 10% of STEC O157:H7 isolated from clinical and bovine samples, respectively, from western Canada were multidrug resistant. Antibiotic resistance in animal- and food-derived strains might increase the risk to humans by food chain or environmental contamination (5). In addition, antibiotic treatment of STEC infections, under certain conditions, increases the release of STEC- derived toxins and the risk of HUS (6). Therefore, it is necessary to develop new alternatives to prevent and treat STEC O157:H7 infections.

As viruses that kill bacteria, the therapeutic value of bacteriophages (phages) in the treatment of bacterial infections has been reassessed over the last 20 years (7). Phages are naturally occurring with low inherent toxicity, are self-replicating, and have high specificity for their bacterial host (8). Phage Ace was found to be efficient against STEC contamination without inducing the release of Stx prophage and Shiga toxin (9). Phage AZO145A was effective in removing STEC O145 biofilms on stainless steel, subsequently significantly reducing the contamination of beef (10). In mouse models of STEC O157:H7 infection, phage therapy was able to reduce the density of E. coli in the gut without distorting the gut microbiota compared to ampicillin treatment (11). However, the effect of phages and antibiotics on the recovery of mammalian host in the treatment of STEC O157:H7 has not been comprehensively analyzed. As one of the promising alternatives to antibiotics, the influence of phages on their mammalian host and on bacterial disease needs to be carefully studied. Here, we systematically compared the therapeutic effects of a three-phage cocktail (PNJ1902, PSD2001, and PSD2002) to enrofloxacin in an enteric mouse model with STEC O157:H7 EDL933. We characterized the impact of the three-phage cocktail and the antibiotic on mouse survival rate, body weight, organ index, composition of gut microbiota, and levels of inflammatory factors.

## RESULTS

### General characteristics of phages.

**FIG 1:**
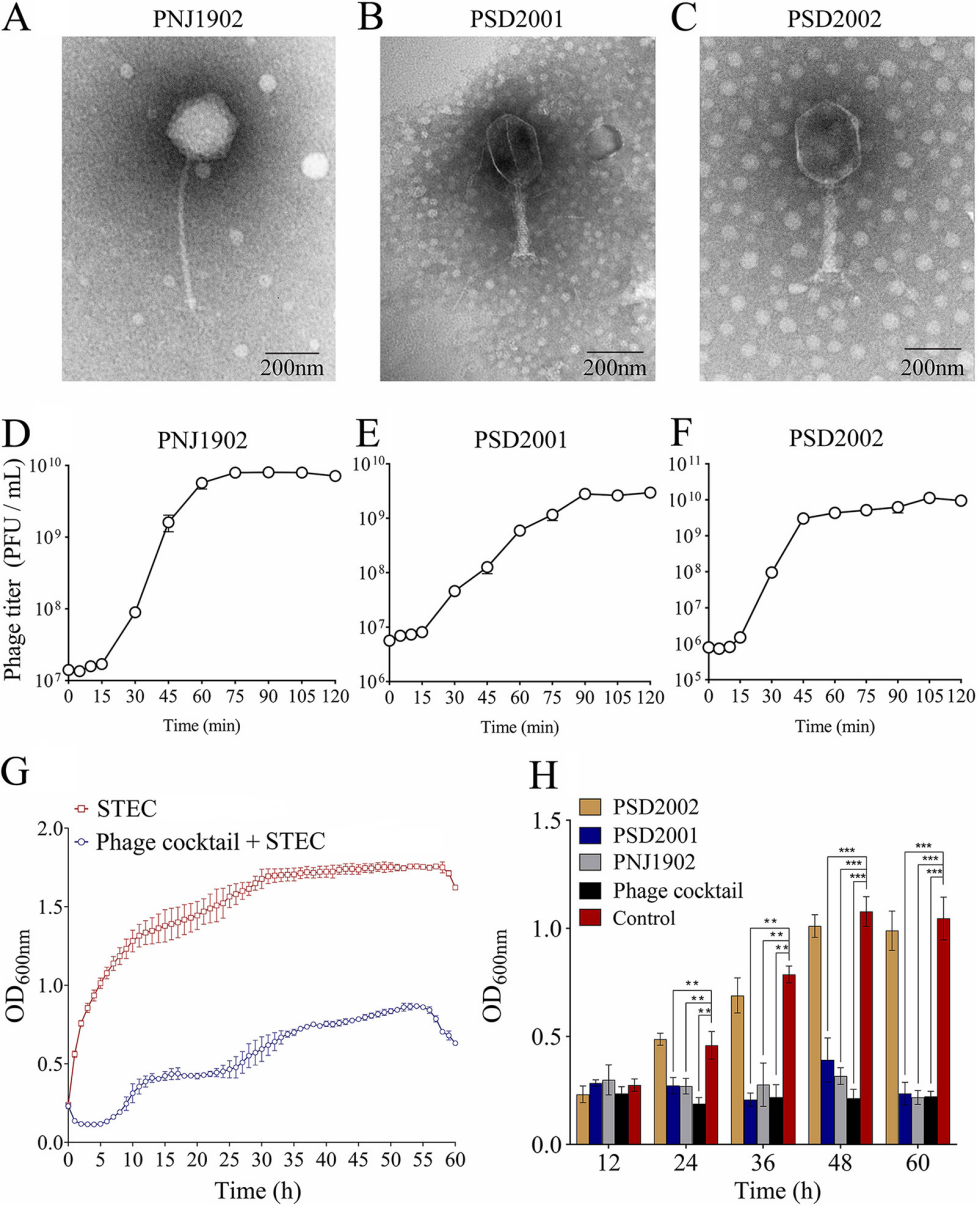
Biological characteristics of the phages. Electron micrographs of phage PNJ1902 (A), PSD2001 (B), and PSD2002 (C). The scale bar represents 200 nm. One-step growth curve of the phage PNJ1902 (D), PSD2001 (E), and PSD2002 (F). Samples (MOI = 0.1) were incubated at 37°C and tested every 5 min for 15 min and every 15 min later (G) Lysis kinetics of STEC O157:H7 EDL933 infected with phage cocktail. The STEC O157:H7 EDL933 was incubated with LB (red frames), or with phage cocktail (blue circles), at 37°C and OD_600nm_ readings were taken each hour. An MOI of 0.1 was used for all conditions. (H) Clearance of STEC O157:H7 EDL933 biofilms *in vitro* with the phage cocktail. 12-h, 24-h, 36-h, 48-h, and 60-h-old biofilms of STEC O157:H7 EDL933 were exposed for 24 h to LB (negative control) or to 10^7^ PFU/mL of phages (single and cocktail). Biofilm biomass was determined by crystal violet staining and quantified by the value of OD_600_. Experiments were performed in triplicate, with *n* =10 per set of conditions, **, *P ≤ *0.01; ***, *P ≤ *0.001. The values indicated are the means of three independent experiments.

The phage cocktail in this study was composed of three phages: PNJ1902, PSD2001, and PSD2002. PNJ1902 belongs to the *Siphoviridae* family, while PSD2001 and PSD2002 belong to the *Myoviridae* family (Fig. 1A to C). We investigated the biological characteristics of the three phages. The results show that the optimal multiplicity of infection (MOI) of phage PNJ1902, PSD2001, and PSD2002 was 0.1, 1, and 10, respectively (Fig. S1A to C). One step growth curves show that the latent period of phage PNJ1902, PSD2001, and PSD2002 was approximately 15 min, 15 min, and 10 min, respectively (Fig. 1D to F). It took approximately 75 to 90 min for the phages to reach the growth plateau phase, resulting in burst sizes of 502, 346, and 635, respectively (Fig. 1D to F). All three phages survived well in pH 4 to 11, with sharp decreases in the phage titer at pH 3 and 12 (Fig. S1D to F). Phages were stable up to 50°C, with variable decreases in titer observed at 60°C and 70°C (Fig. S1G to I). The cocktail significantly inhibited the growth of STEC O157:H7 EDL933 up to 60 h *in vitro* (Fig. 1G). We also measured the biofilm clearance efficiency of the phage cocktail on already formed biofilms, and observed considerable removal of 24-h, 36-h, 48-h, and 60-h-old biofilms compared with the control (*P < *0.01) (Fig. 1H). Both phage PNJ1902 and PSD2001 individually showed high biofilm clearance efficiency, although this was not as high as the cocktail. These results suggest that this phage cocktail has a potential antimicrobial effect against STEC O157:H7 EDL933 infection.

### Genome analysis of the three phages.

Genome assemblies of all three phages were obtained using the Unicycler assembly. The size of the genomes ranges from 107.5 Kbp to 172.4 Kbp (Table 1). A BLAST search against the NCBI database revealed that all three phages most closely matched Enterobacter or E. coli phages (Table 1). Annotations showed that all three phage genomes contained both phage structural and replication features and lacked integrase and recombination genes, with lytic phage properties observed in VIBRANT analysis. None of the genomes contained antibiotic resistance and putative virulence genes as predicted by ABRicate. Hence, our genome analysis strongly suggests that the phages possess desirable properties for phage therapy.

**TABLE 1:** Genome information of the three phages

Phage	Genome size (bp)	GC content (%)	ORF^*a*^	Best match (NCBI database)	GenBank accession #
PNJ1902	107,506	39.0	197	Salmonella phage vB_SenS_S124 (GenBank Accession #: OK108607.1) (93% coverage and 97.6% identity)	OK254197.1
PSD2001	172,175	44.4	287	Escherichia phage P479 (GenBank Accession #: MW269952.1) (92% coverage, 97.1% identity).	OK254198.1
PSD2002	172,439	39.5	274	Escherichia phage WG01 (GenBank Accession #: NC_031928.1) (95% coverage and 98.4% identity)	OK335775.1

a Based on RAST annotation.

### The phage cocktail exhibited comparable efficacy with enrofloxacin in treating STEC O157:H7 EDL933 infection.

As it was previously reported that phage-induced endotoxin release could be an issue during phage therapy (12), the concentration of endotoxin released by STEC O157:H7 after being treated by the phage cocktail or enrofloxacin *in vitro* was tested. The results show that the bacterial endotoxin content caused by the phage cocktail was not significantly different (*P > *0.05) to that caused by enrofloxacin (Fig. S2).

**FIG 2:**
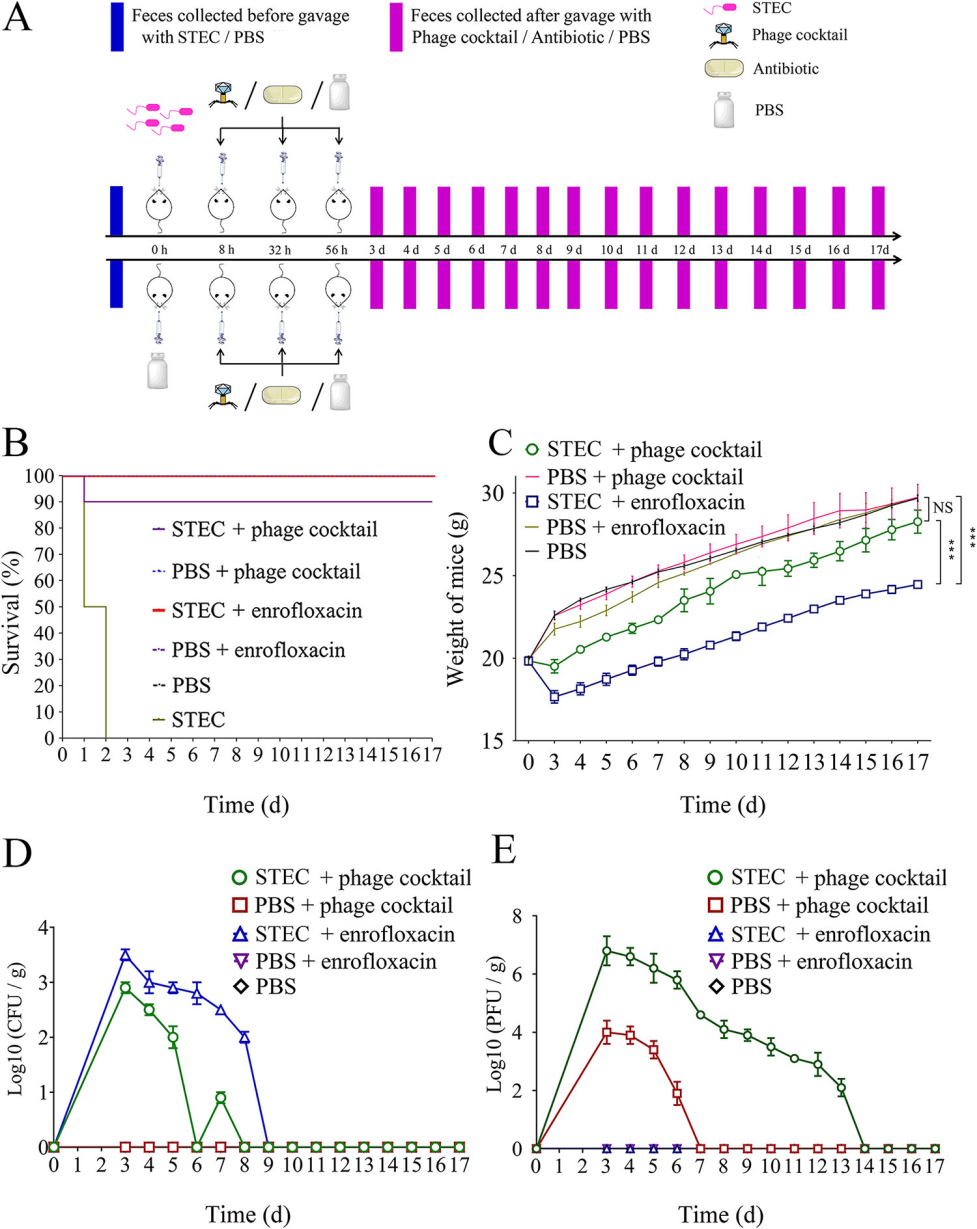
Experimental design and health status of mice in each group. (A) Flow chart of gavage of mice in each group. (B) Overall survival. The number of mice in each group is 10. For each group of mice, the results shown are from two independent experiments. (C) Weight change of the mice in each group. (D) Monitoring of the number of STEC O157:H7 EDL933 bacteria in mice feces. (E) Monitoring of the number of phages in mice feces.

Prior to challenge, we screened the feces of the mice for the presence of STEC O157:H7 EDL933 and the three phages. The results showed that the no phage or STEC O157:H7 EDL933 were recovered prior to inoculation in the mice gut. Mice were then orally challenged with a high dose of STEC O157:H7 EDL933 (10^9^ CFU/mL) leading to a 100% lethality rate 2 days postinfection. Eight hours postinfection, mice showed clinical symptoms. At this time point, bacteria challenged mice were treated with either the phage cocktail (10^8^ PFU/mL) or enrofloxacin (10 mg/kg). Enrofloxacin treatment resulted in 100% (10/10) survival rates, while phage cocktail treatments resulted in 90% (9/10) survival rates based on two independent experiments (Fig. 2B). Survival rates of 100% were obtained in the three control groups: phosphate buffered saline (PBS) + phage, PBS + enrofloxacin, and PBS. Surprisingly, while the enrofloxacin treatment group showed higher survival rates than the phage cocktail treatment group, the enrofloxacin treatment group exhibited a greater weight loss (*P < *0.001) than the phage cocktail treatment group (Fig. 2C). We observed that while the two groups presented a similar tendency to recover weight, the weight of the phage cocktail treatment group was closer to that of the PBS control group than to that of the enrofloxacin treatment group on day 17 (*P* < 0.001) (Fig. 2C).

### Phage therapy led to less organ toxicity than enrofloxacin treatment.

Organ weight change is an important endpoint used by regulatory agencies to develop toxicity reference values for use in human health risk assessments (13). The change in organ index (relative organ weight) often better reflects the toxicity to the organs in comprehensive situations (14). To further analyze the impacts of toxic substances on animal immunity and metabolism, we quantified the body weight and organ index of each group. The results show that there were no significant differences in body weight or the organ index between the STEC + phage cocktail group, PBS + phage cocktail group, PBS + enrofloxacin, and the PBS group (*P > *0.05) (Table 2). However, the body weight of mice in the STEC + enrofloxacin group was significantly lower than that of the control group (PBS group) (*P < *0.01) (Table 2). Moreover, the liver, spleen, heart, lung, muscle, and brown adipose tissue (BAT) weights were significantly reduced (*P < *0.05), leading to significant reductions (*P < *0.05) in the organ indexes of the liver, spleen, and BAT, and a significant increase (*P < *0.05) in the organ index of the kidney (Table 2). These results indicate that compared with antibiotics, the phage cocktail treatment effectively maintained body weight stability and was less toxic to organs.

**TABLE 2:** General characteristics of mice in different groups^*a*^

Characteristics	PBS	STEC + phage cocktail	PBS + phage cocktail	STEC + enrofloxacin^*b*^	PBS + enrofloxacin
Body wt (g)	29.68 ± 0.19	28.26 ± 0.7	29.74 ± 0.69	24.46 ± 0.23^*c*^▾	29.73 ± 0.16
Liver (g)	1.80 ± 0.12	1.73 ± 0.13	1.75 ± 0.12	1.23 ± 0.11^*d*^▾	1.69 ± 0.10
Kidney (g)	0.39 ± 0.03	0.40 ± 0.06	0.38 ± 0.04	0.36 ± 0.02	0.38 ± 0.04
Spleen (g)	0.18 ± 0.02	0.16 ± 0.02	0.18 ± 0.03	0.12 ± 0.01^*d*^▾	0.17 ± 0.02
Heart (g)	0.30 ± 0.01	0.27 ± 0.02	0.29 ± 0.01	0.22 ± 0.01^*d*^▾	0.28 ± 0.02
Lung (g)	0.38 ± 0.03	0.37 ± 0.02	0.39 ± 0.02	0.30 ± 0.01^*d*^▾	0.39 ± 0.01
Muscle (g)	0.34 ± 0.03	0.32 ± 0.02	0.33 ± 0.02	0.28 ± 0.04^*d*^▾	0.33 ± 0.03
BAT (g)	0.10 ± 0.01	0.10 ± 0.01	0.11 ± 0.02	0.06 ± 0.01^*d*^▾	0.10 ± 0.02
Relative liver wt (%)	6.06 ± 0.52	6.12 ± 0.34	5.88 ± 0.48	5.03 ± 0.38^*d*^▾	5.68 ± 0.46
Relative kidney wt (%)	1.31 ± 0.1	1.42 ± 0.09	1.28 ± 0.08	1.47 ± 0.12^*d*^▴	1.28 ± 0.11
Relative spleen wt (%)	0.61 ± 0.08	0.57 ± 0.09	0.61 ± 0.07	0.49 ± 0.07^*d*^▾	0.57 ± 0.06
Relative heart wt (%)	1.01 ± 0.06	1.00 ± 0.08	1.00 ± 0.04	0.9 ± 0.07	0.94 ± 0.08
Relative lung wt (%)	1.28 ± 0.11	1.31 ± 0.08	1.31 ± 0.08	1.23 ± 0.12	1.31 ± 0.13
Relative muscle wt (%)	1.15 ± 0.06	1.13 ± 0.07	1.11 ± 0.05	1.14 ± 0.08	1.11 ± 0.08
Relative BAT wt (%)	0.34 ± 0.03	0.35 ± 0.05	0.37 ± 0.07	0.25 ± 0.04^*d*^▾	0.34 ± 0.06

a Data are expressed as mean ± SD, *n* = 5 mice/group.

b The ▾ and ▴ indicate that the data decreases or increases. The data of different groups were detected at 17th day.

c The difference is significant extremely significant (*P* < 0.01).

d The difference is significant (*P *< 0.05).

### Phage eliminated pathogenic E. coli *in vivo* faster than enrofloxacin.

To compare the elimination efficiencies of the phage cocktail and enrofloxacin on pathogenic bacteria *in vivo*, we looked at the decrease in STEC O157:H7 EDL933 over time in mice feces. Three days following treatment with the phage cocktail (day 6), the level of STEC O157:H7 EDL933 sharply dropped below the detection threshold (Fig. 2D). Although a small recovery on day 7 was seen, STEC O157:H7 EDL933 was eliminated by the phages by day 8 (Fig. 2D). Conversely, the level of STEC O157:H7 EDL933 in the antibiotic treatment group decreased at a slower rate than the phage treatment group during the first 8 days, with STEC O157:H7 EDL933 finally removed by day 9 (Fig. 2D). We next investigated the replication of phages in mice feces. Fecal phage titer decreased strongly in the group of mice which did not receive bacteria and was undetectable by day 7 (Fig. 2E). By contrast, the fecal phage titer in mice colonized with STEC O157:H7 EDL933 was two logs higher over a period of 2 weeks (Fig. 2E). These results suggest that intestinal colonization of STEC O157:H7 EDL933 supported long-term phage replication in mice. Phage resistance in STEC O157:H7 EDL933 *in vivo* was tested. Among 200 colonies randomly selected, only 4% (8/200), 1.5% (3/200), and 2.5% (5/200) of the colonies were resistant to phage PSD2001, PSD2002, and PNJ1902, respectively, indicating that the resistance rate *in vivo* was very low, which leads to efficient elimination of STEC by these phages.

### Both phages and enrofloxacin could alleviate intestinal injury.

**FIG 3:**
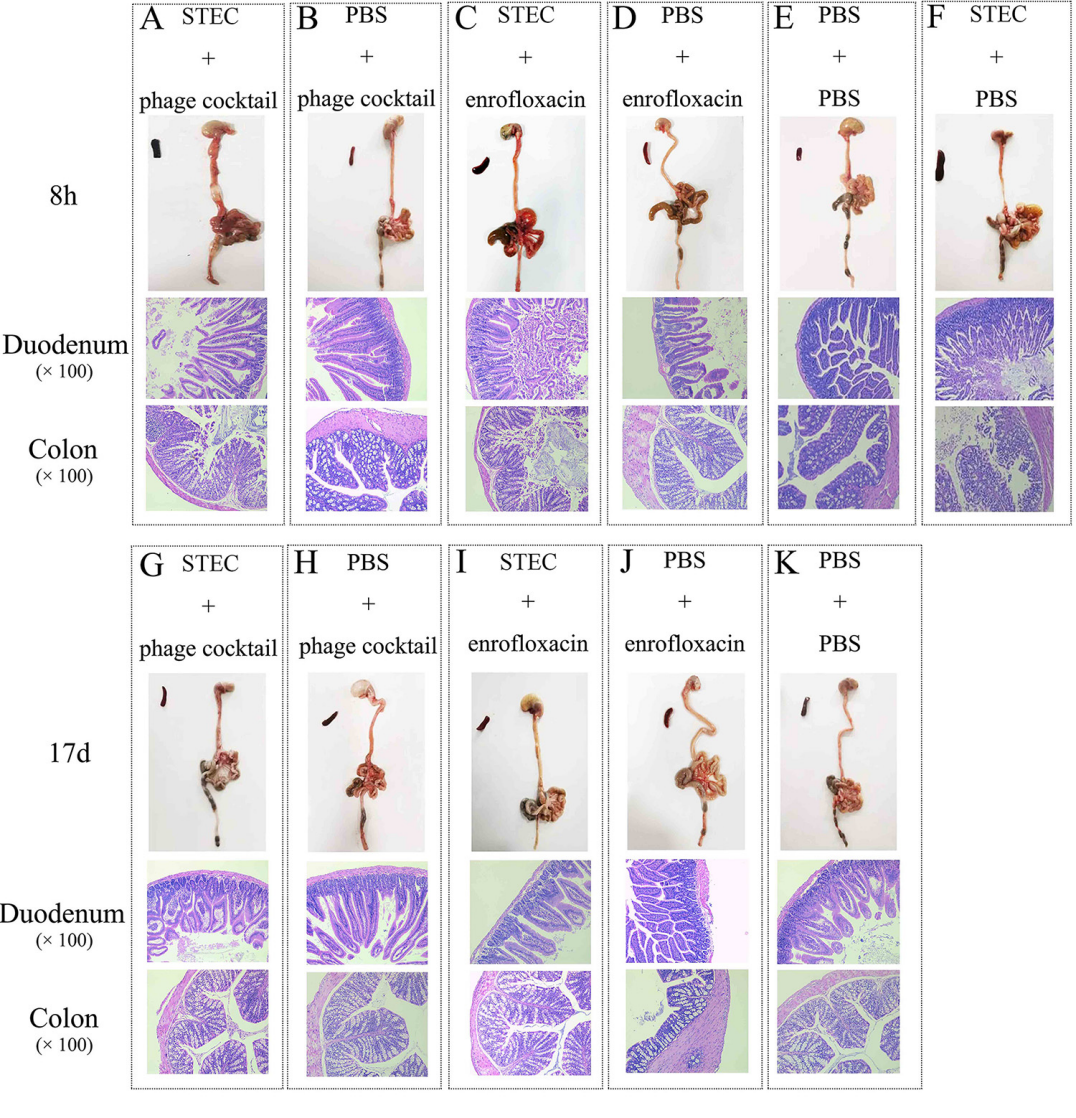
HE staining of mouse gut. HE stained of mouse duodenum and colon tissues at 8h of STEC + phage cocktail group (A), PBS + phage cocktail group (B), STEC + enrofloxacin group (C), PBS + enrofloxacin group (D), PBS + PBS group (E) and STEC + PBS group (F). And HE stained of mouse duodenum and colon tissues at 17d of STEC + phage cocktail group (G), PBS + phage cocktail group (H), STEC + enrofloxacin group (I), PBS + enrofloxacin group (J), PBS + PBS group (K). The magnification of mouse duodenum and colon tissues is ×100.

To investigate the pathological changes in the intestines of mice across different groups, duodenum and colon tissues were sliced and hematoxylin and eosin (H&E) stained at 8 h and 17 days postinfection. A representative image from each group of five mice is presented. Compared with control mice (Fig. 3E), the digestive systems of the diseased mice showed obvious thinning of the intestinal wall, gastrointestinal swelling, and hyperemia (Fig. 3F). The gut villi of the duodenum and colon tissues of the mice were severely damaged 8 h after challenge with STEC O157:H7 EDL933 (Fig. 3A, C, and F). After treatment with the phage cocktail or enrofloxacin, the gut villi of each treatment group were almost completely recovered compared to the control groups (Fig. 3G to K). The damage to the gut villi was further quantified by Chiu^’^s analysis. The results showed that, compared with the PBS control group, STEC O157:H7 EDL933 caused serious damage to the gut villi of mouse duodenum and colon tissues (*P < *0.0001), while the phage and enrofloxacin treated groups did not exhibit significant intestinal tissue damage (*P > *0.05) (Fig. S3). Chiu^’^s analysis also demonstrated that there was no significant difference in the degree of intestinal injury in mice between the phage cocktail and enrofloxacin treated groups (*P* >0.05) (Fig. S3).

### The level of inflammatory factors in phage treated mice recovered faster than in the enrofloxacin group.

**FIG 4:**
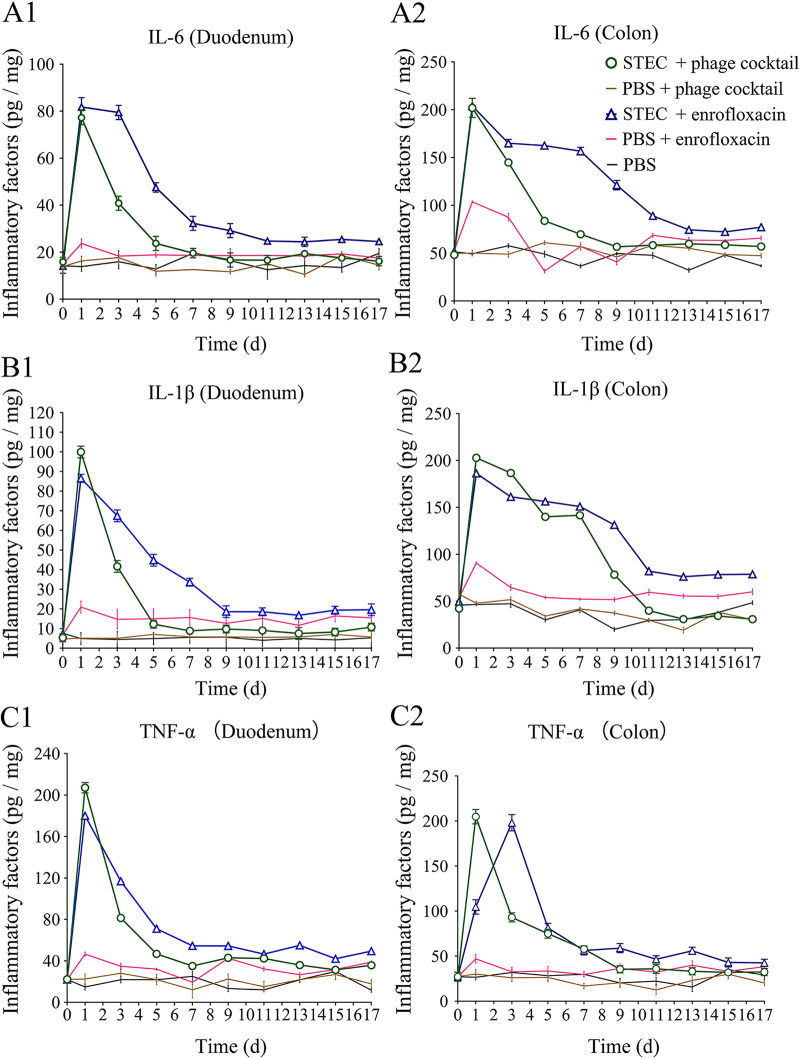
Levels of inflammatory cytokines IL-6, IL-1β, and TNF-α in intestine. The concentration of inflammatory cytokines IL-6, IL-1β, and TNF-α in the duodenum and colon of mice were detect by ELISA. The concentration of IL-6 in the duodenum (A1) and colon (A2) of mice. The concentration of IL-1β in the duodenum (B1) and colon (B2) of mice. The concentration of TNF-α the duodenum (C1) and colon (C2) of mice.

It has been reported that IL-1β and IL-6 are widely involved in many pathological injury processes, such as tissue destruction and edema formation, and the levels of these proinflammatory cytokines thus broadly reflect the presence of injury and inflammation (15). As expected, STEC O157:H7 EDL933 induced high levels of the inflammatory factors IL-1β, IL-6 and TNF-α, which were significantly increased on the first day (*P < *0.05) (Fig. 4). However, with the exception of IL-1β in the colon, the levels of all inflammatory factors in the STEC + phage cocktail group decreased sharply on the fifth day and remained relatively stable during the following 12 days (Fig. 4A1, A2, B1, C1, and C2). In contrast, the levels of most inflammatory factors in the duodenum and colon of mice in the STEC + enrofloxacin group decreased much more slowly during the first week and remained at higher levels relative to the STEC + phage cocktail group (Fig. 4A1, A2, B1, and C1). It is noteworthy that even enrofloxacin on its own (PBS + enrofloxacin) resulted in a moderate induction of inflammatory factors. The level of IL-1β in the PBS + enrofloxacin control group even remained higher than in the PBS + phage cocktail and PBS groups during the whole period (Fig. 4B1 and B2). These results indicate that phage treatment exhibited reduced inflammation compared to antibiotics.

### The phage treatment group released less endotoxin and Shiga toxin than the enrofloxacin treatment group *in vivo*.

The endotoxin and Shiga toxin released by STEC O157:H7 EDL933 in mice sera after being treated with the phage cocktail or enrofloxacin was determined. The results show that both phage and enrofloxacin treatment significantly reduced endotoxin and Shiga toxin release compared with that of the STEC challenge group. However, the phage treatment group released much less endotoxin and Shiga toxin than the enrofloxacin treatment group (Fig. S4).

### The phage treatment group exhibited less disturbance of the gut microbiota than the enrofloxacin group.

One of the advantages of phages over antibiotics is their narrow host range, leading to a low impact on the microbiota composition (16). Here, we compared the fluctuation of the microbiota in the guts of mice treated with the phage cocktail or enrofloxacin according to Yang’s method (17). A total of 10 bacterial taxa were monitored, including *Candidatus Saccharibacteria*, *Delta-* and *Gammaproteobacteria*, *Deferribacteres*, *Betaproteobacteria*, *Epsilonproteobacteria*, *Actinobacteria*, *Verrucomicrobia*, *Bacteroidetes*, *Tenericutes*, and *Firmicutes*.

**FIG 5:**
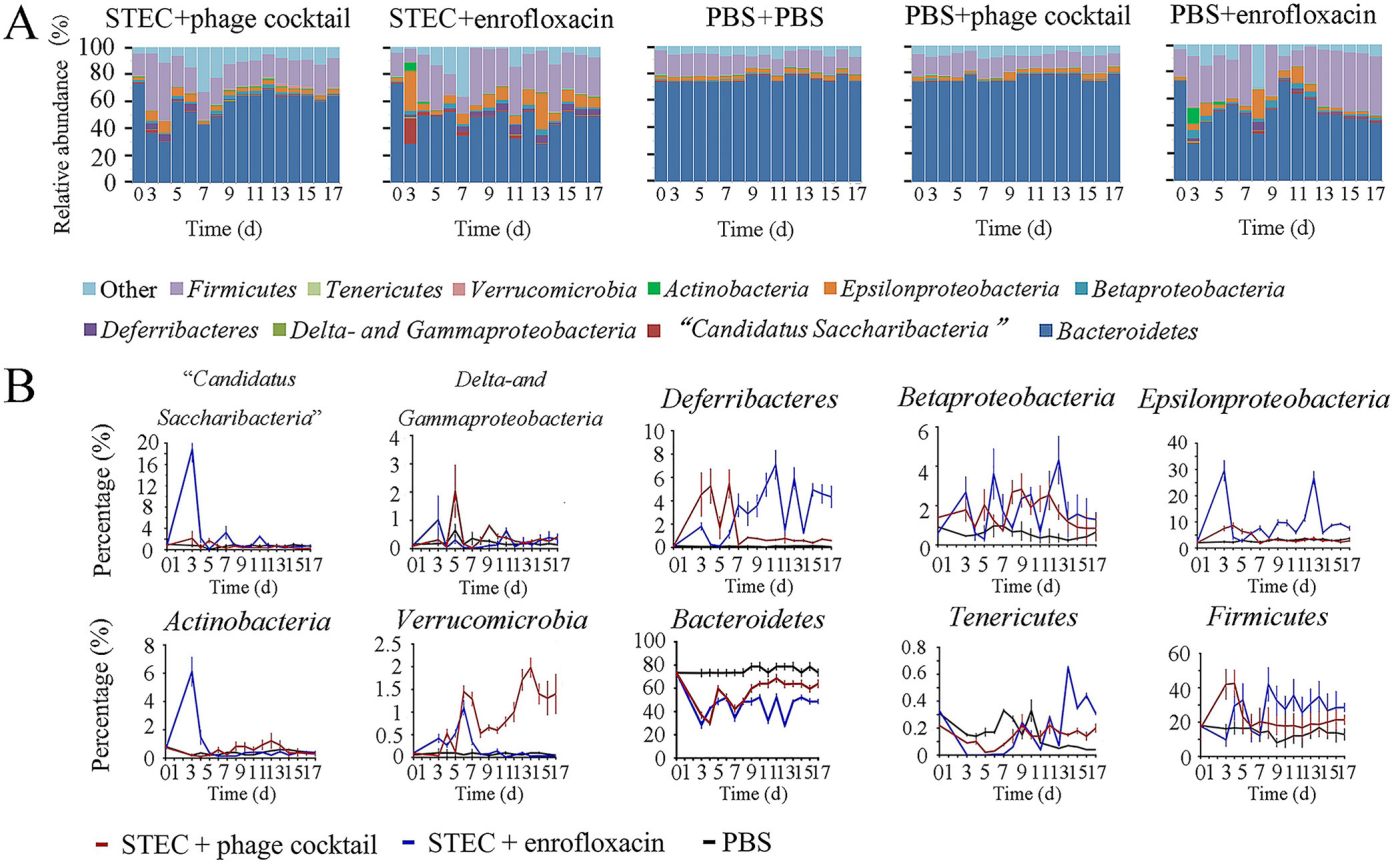
Analysis of the abundance of gut microbiota in mice. (A) Dynamic monitoring of the relative abundance of the mice gut microbiota of phyla in each group. (B) Daily relative abundance of each bacterial taxa in the STEC + phage cocktail group, STEC + enrofloxacin group, and PBS control group.

We observed no significant difference in the proportion of each taxon in the gut microbiota of mice in each group at 0 h. After infection and three consecutive doses of either phage or antibiotic, the microbiota compositions of both treatment groups exhibited differences. It is important to note that the fluctuation in the relative abundance of different phyla in the enrofloxacin treated group was higher than the phage treated group. Subsequently, after approximately 1 week, the microbiota compositions of STEC + phage cocktail group recovered to a relatively stable state, which was similar to the PBS control group (Fig. 5A). On the contrary, the STEC + enrofloxacin group seemed to undergo 2 weeks’ fluctuation in microbiota compositions, before reaching a stable state that was different from the PBS control group (Fig. 5A).

When comparing the final microbiota compositions on day 17 with the initial day 0 population, we found that the relative abundance of the gut microbiota of mice in the STEC + enrofloxacin group fluctuated significantly on the 17th day (*P < *0.05) (Fig. 5A). *Bacteroidetes* and *Firmicutes* are two abundant taxa that comprised more than 89% of the microbiota in all groups of mice on day 0, which is similar to humans (18, 19). The proportion of *Firmicutes* increased from 17.7% to 28.6%, while the proportion of *Bacteroides* dropped from 73.0% to 48.7%, in the STEC + enrofloxacin group (Fig. 5A). However, the STEC + phage cocktail group exhibited a much smaller change in the fluctuation of the microbiota: the proportion of *Firmicutes* increased from 16.6% to 21.4%, and the proportion of *Bacteroides* dropped from 73.3% to 63.9% (Fig. 5A). In addition, the enrofloxacin-only gavage caused a significant fluctuation in the gut microbiota, in which the proportion of *Firmicutes* increased from 19.90% to 43.4%, and the proportion of *Bacteroides* dropped from 73.2% to 42.5%. Comparatively, the phage-only treatment did not cause any significant disturbance to the gut microbiota (Fig. 5A), indicating that phage interferes less with the gut microbiota than antibiotics.

We next analyzed the changes in relative abundance of each bacterial taxon during the 17 days. The fluctuation of *Candidatus Saccharibacteria*, *Epsilonproteobacteria*, and *Actinobacteria* in the STEC + phage cocktail group was very low, remaining quite close to the control group, while the relative abundance of three other phyla in the STEC + enrofloxacin group significantly increased during the first 3 days, which was followed by a sharp decrease on the fourth day (Fig. 5B). For *Deferribacteres*, *Betaproteobacteria*, *Bacteroidetes*, *Tenericutes*, and *Firmicutes*, both the STEC + enrofloxacin and the STEC + phage cocktail group exhibited fluctuations (Fig. 5B). The only exception was *Verrucomicrobia*, which was significantly increased in the STEC + phage cocktail group, while quite stable in the STEC + enrofloxacin and control group (Fig. 5B).

## DISCUSSION

While growing evidence demonstrates that phage therapy has clinical utility in treating various antibiotic-resistant bacterial infections, the efficacy of phage therapy in the treatment of enteric bacterial infections remains largely unknown (20). Here, we compared the therapeutic efficacy of a phage cocktail versus an antibiotic (enrofloxacin) for the treatment of STEC O157:H7 EDL933 enteric infections in a mouse model. We observed that phages had significant advantages over antibiotics for the recovery of treated mice. These included an improved recovery in body weight and reduced organ toxicity caused by pathogenic bacteria; improved elimination of bacterial load from the intestine; decreased inflammatory factors IL-1β, IL-6 and TNF-α; and reduced fluctuations in the composition of gut microbiota. These results highlight the potential benefits of phage therapy in combating enteric infections over antibiotics. Taken together, these results suggest that phages have great potential in the treatment of gut infections.

Previously, many researchers believed that the lysis of bacteria by phages would lead to the production of endotoxin, which would cause a strong immune response in the host body (8). Conversely, other studies have shown that therapeutically relevant phages, characterized by low endotoxin release and rapid bactericidal effects, are non-inferior to β-lactams (12). Some studies have reported that antibiotic treatment of STEC infections, under certain conditions, increased the release of STEC-induced toxins and increased the risk of HUS (6). To the contrary, some studies have reported that a good therapeutic effect was obtained by antibiotic treatment of STEC infections (21). The aim of our research was to compare the therapeutic effect of phage and antibiotic treatment. To exclude the effects of different amounts of endotoxin on the body, we compared several antibiotics and finally chose enrofloxacin, which leads to a similar amount of endotoxin release *in vitro* to that of phage (Fig. S2). Then we measured the amount of both endotoxin and Shiga toxin release *in vivo* (in mice sera) after phage and antibiotic treatment. The results show that both phage and antibiotic treatment significantly reduced endotoxin and Shiga toxin release relative to the STEC challenge group, with no detection after 7 days. However, in contrast to the results *in vitro*, enrofloxacin treatment released a much higher level of endotoxin and Shiga toxin than that of the phage treatment group (Fig. S4). We speculate that enrofloxacin induced more Shiga toxin, which resulted in damage to enterocytes, leading to more endotoxin being released into the serum through the damaged intestinal mucosa (22). In addition, due to the narrow host spectrum of the phages, phage treatment would have specifically killed only STEC O157:H7 in the gut, whereas enrofloxacin treatment would have killed a large number of bacteria, resulting in more endotoxin being released in the enrofloxacin treatment group. Considering the complex ecological environment in the mouse gut reduces the frequency of phage-pathogen contact (23), further studies are required.

In most previous studies, phages were administered to animals at either the same time or shortly after the pathogen was administered (1h to 4 h) (24, 25), which might prevent the manifestation of the majority of clinical symptoms. This led us to question the practical value of such short treatment administration times. In real-world clinical scenarios, phages are much more likely to be administered after clinical symptoms have emerged. As such, to mimic the clinical scenario more closely, we treated mice with phages 8 h after bacterial challenge, and importantly, after mice had developed clinical signs (including hunched backs and ruffled hair). In addition, histopathological and Chiu's analysis of the duodenums and colons of the mice demonstrated that the gut villi were damaged 8 h after bacterial challenge.

The survival rate of the phage treated group was 10% lower than that of the enrofloxacin treated group. It should be noted that the dose of enrofloxacin (10 mg/kg) was obtained from our pre-experiment to ensure 100% rescue of lethally infected mice in order to avoid any bias in favor of phages. Although the use of enrofloxacin provided 100% protection of mice in our study, the increased risk of HUS development with quinolone antibiotics must not be ignored (26). The dose of the phage cocktail (10^8^ PFU/mL) was based on the highest titer obtained when replicated in host bacteria in LB media. It has been shown that the therapeutic effect of phage therapy is sometimes dose-dependent and if we increase our phage dose, or further increase the frequency of phage administration (27), higher protection rates may be obtained.

One prominent advantage of phage therapy is its high specificity and rapid lysis of host bacteria. Thus, in this study STEC O157:H7 EDL933 load in the phage therapy group decreased much faster than the enrofloxacin therapy group. After 2 days of treatment with the phage cocktail, the E. coli load in mice feces decreased below 10^2^ CFU/mL, while the bacterial load of the enrofloxacin therapy group decreased slowly and remained above 10^2^ CFU/mL during the first 4 days after treatment. As such, the improved recovery in terms of weight gain, inflammatory markers and gut microbiota seen in the phage treated group was likely due in part to the fast elimination of pathogenic bacteria.

The gut microbiota is important for our overall health and well-being (28, 29). Phage host specificity leads to lower impact on the microbiota composition. A recent study by Galtier et al. (16) reported that antibiotic treatment led to a significant decrease in the abundance of 50 genera and an increase in 14 genera, while only 11 genera decreased and 21 genera increased after phage treatment. In the present study, the phage cocktail on its own had no observable impact on the gut microbiota in mice. The consequences of disturbances in the gut microbiota are often non-directional (30, 31). It leads us to worry about whether it will cause harmful bacteria to become dominant in the microbiota when using different antibacterial agents. In the gut microbiota, *Firmicutes* and *Bacteriodetes* account for approximately 90% of the phylum level (17). Moreover, a change in the ratio of *Firmicutes*/*Bacteriodetes* has a great impact on the body’s health. Studies have shown that an increase in the *Firmicutes*/*Bacteriodetes* ratio is related to metabolic syndrome and obesity (32, 33). Here, we found that the ratio of *Firmicutes*/*Bacteriodetes* of mice in the antibiotic treatment group increased from 0.2 to 0.6, while the ratio of *Firmicutes*/*Bacteriodetes* of mice treated with the phage cocktail only slightly increased from 0.2 to 0.3. This suggests that compared with antibiotics, phages could help rebuild a healthier gut microbiome after treating gut diseases.

### Conclusion.

With the increasing frequency of antibiotic-resistant strains of pathogenic bacteria, urgent measures need to be taken to find alternative treatments. Among putative solutions, phage therapy is considered a promising alternative (34). To evaluate whether phages can replace antibiotics against enteric colibacillosis, we comprehensively compared the therapeutic effect of a phage cocktail with enrofloxacin on STEC O157:H7 EDL933 infection for the first time. Our results reveal that while our phage cocktail was not as efficient as antibiotics in treating STEC infections, it was superior with respect to mouse recovery, with reduced gut inflammation, increased body weight gain, alleviation of organ toxicity, and maintenance of gut microbiota homeostasis. Our findings demonstrate significant benefits of phages compared with antibiotics, which represents a completely new approach to the treatment of inflammatory bowel disease caused by bacteria.

## MATERIALS AND METHODS

### Bacterial strain and growth conditions.

The STEC O157:H7 EDL933 strain was originally isolated from raw hamburger meat and produced Shiga-like toxins I and II. Bacteria were cultured in Luria-Bertani (LB) liquid media or LB agar (2% agar) plates at 37°C.

### Phage isolation, preparation, and characterization.

Phages PNJ1902, PSD2001, and PSD2002 of STEC O157:H7 EDL933 were isolated from the feces of chicken farms. The morphology, pH and temperature resistance, optimal MOI, and one-step growth curve for each phage were determined by standard methods (see details in the Supplemental File 1). The phage cocktail was prepared by mixing equal numbers of the three phages PNJ1902, PSD2001, and PSD2002. In this study, the STEC O157:H7 EDL933 strain was used for plaque assays of phages PNJ1902, PSD2001, and PSD2001 alone or the phage cocktail.

### *In vitro* lysis kinetics.

STEC O157:H7 EDL933 was cultured in LB liquid medium until the OD_600nm_ = 1.0, which corresponds a concentration of 10^8^ CFU/mL. The concentration of the phage cocktail was adjusted to 10^7^ PFU/mL. One-hundred μL of the bacterial culture were mixed with 100 μL of the phage cocktail or LB liquid medium in a 96-well plate, which was then incubated in a microplate reader (TECAN, Switzerland) at 37°C with orbital shaking. Two-hundred μL of LB liquid were added to the 96-well plate as a blank control. OD_600nm_ values were recorded automatically each hour for 60 h.

### Biofilm clearance assay.

The biofilm clearance assay was performed as previously described with minor modifications (35). The biofilms of STEC O157:H7 EDL933 were cultured on 96-well plates for 12 h, 24 h, 48 h, and 60 h, washed with PBS three times to remove planktonic bacteria and medium, and then treated with 200 μL of phage solution (10^7^ PFU/mL) for 24 h. The removal of bacterial biofilm by the phage cocktail was determined by the crystal violet staining method. The biofilm was quantified by measuring the OD_600nm_ value using a spectrophotometer (Eppendorf, Germany).

### Endotoxin and Shiga toxin detection.

The endotoxin released by STEC O157:H7 EDL933 after being treated with the phage cocktail or enrofloxacin *in vitro* was measured as previously described (12). Released free endotoxins were tested using the ToxinSensor Color Limulus Endotoxin Detection Kit (Genscript, China), according to the manufacturer’s instructions. A standard curve for each test was constructed and the concentration of endotoxin was determined according to the standard curve. The assays were independently repeated three times. The endotoxin and Shiga toxin released by STEC O157:H7 EDL933 *in vivo* after being treated with the phage cocktail or enrofloxacin was also determined. The sera of the infected mice treated with the phage cocktail or enrofloxacin were collected on days 0 to 7. The concentrations of endotoxin and Shiga toxin in the sera were measured according to the manufacturer’s instructions using an Endotoxin kit (Limulus reagent method) (BIOENDO, China) and E. coli O157 Shiga Toxin ELISA Kit (mlbio, China), respectively.

### Genome sequencing and analysis.

The phage genomic DNA was extracted with λ phage genomic DNA extraction kit (ABigen, China), dissolved in ddH_2_O, and stored at −20°C. The phage DNA was sent to Novogene Bioinformatics Technology Co., Ltd. (Tianjin, China) for whole-genome sequencing. The Illumina NovaSeq 6000 sequencing platform was used and the NEBNext Ultra DNA Library Prep Kitfor Illumina Kit (NEB, USA) was used to construct the DNA library. Raw sequencing reads of the phage genomes for phages PNJ1902 (SRR18692579), PSD2001 (SRR18692585), and PSD2002 (SRR18692629) have been uploaded to NCBI SRA. In addition, raw reads were quality filtered using Trimmomatic v 0.39 to remove adaptor sequences and reads with a quality of less than 15 in a sliding window of 4 bp (36). Raw reads were then mapped to the genome of the host of isolation (STEC O157:H7 EDL933) to filter out any carry over contamination of the bacterial genome using BBDuk, a tool in the BBmap package (37). The filtered raw reads were assembled using Unicycler v 0.43 with a flagged minimum contig length of 1,000 bp (38). The assembled genomes were annotated using the RAST sever v 2.0 (39). The phage genomes were also examined for lytic characteristics using VIBRANT and for the presence of antibiotic resistance and virulence genes using various databases of resistance and virulence gene finder implemented in ABRicate (https://github.com/tseemann/abricate) (40).

### Ethics statement.

All procedures using SPF ICR mice (Institute of Cancer Research/USA; Institute of Comparative Medicine, College of Veterinary Medicine, Yangzhou University, China) were approved by the Association for Assessment and Accreditation of Laboratory Animal Care International, and all animal experiments were performed after approval by the Ethical Committee for Animal Experiments of Nanjing Agricultural University (PTA2019024), Nanjing, China.

### Animal infection and treatment.

To eliminate the interference of phage or bacteria carried by mice on the results of this experiment, the presence of STEC O157:H7 EDL933 and the phages PNJ1902, PSD2001, and PSD2002 were determined in mice feces before the experiment. STEC O157:H7 EDL933 was detected by PCR (Table S1) (41, 42) and phages were detected by plaque assay. As shown in Fig. 2A, 60 female SPF ICR mice (4-weeks old) were randomly divided into six groups (10 mice/group): (i) STEC + phage; (ii) STEC + enrofloxacin; (iii) STEC + PBS; (iv) PBS + phage; (v) PBS + enrofloxacin; (vi) PBS + PBS. Before the experiment, all mice were gavaged with 100 μL of NaHCO_3_ (1 M) to neutralize gastric acid. 5 min later, mice were gavaged with 200 μL of E. coli (10^9^ CFU/mL) or PBS. Eight hours later, mice were gavaged with 200 μL of the phage cocktail (10^8^ PFU/mL), enrofloxacin (10 mg/kg) or PBS, according to the group. The treatment was repeated twice with 24 h intervals. Mice were monitored for 2 weeks, and the fecal samples were collected every day.

### Histological analyses.

Five mice were randomly selected in each group and euthanized at 8 h and 17 days postinfection. The duodenum and colon samples were dissected and fixed for paraffin embedding (17). Paraffin-embedded sections with a thickness of 5 μm were stained with H&E. A tissue histological examination was performed under an optical microscope (Olympus, Japan). Chiu’s Pathology score was used to quantify the pathological changes of the intestinal tract (43).

### Weight loss evaluation.

The weights of the mice and their different organs, including the liver, kidney, spleen, heart, lung, muscle tissue, and BAT of each group at 0 h and 17 d, were recorded. Organ coefficients were calculated using the formula: organ coefficient (%) = organ weight (g)/body weight (g) × 100% (44).

### Expression of inflammatory factors.

The concentrations of inflammatory factors of the mice in different groups were measured as previously described (45). The concentrations of IL-6, IL-1β, and TNF-α in the duodenum and colon were measured every 2 days during the experiment using an ELISA kit (NJJCBIO, China). Each test was performed in triplicate.

### Microbiota diversity assessment.

Fresh feces from the mice in each group were collected at 0 days and each day of 3 to 17 days. The microbiota diversity in the guts of the mice was analyzed according to Yang’s method (17). Total DNA was extracted from the fecal samples using a stool DNA kit (Omega, USA). Real-time PCR (RT-PCR) was used to calculate the daily content of 10 bacterial taxa in the guts of the mice. The primers are shown in Table S2. The average C_t_ value obtained from each primer pair was transformed into a percentage using the following formula (17):

$$\text{X}=\frac{{\left(\text{Eff}.\text{Univ}\right)}^{CT_{\text{univ}}}}{{\left(\text{Eff}.\text{Spec}\right)}^{CT_{\text{spec}}}}\times 100$$

“X” represents the percentage of 16S taxon-specific copy number existing in a sample; Eff. Univ and Eff. Spec refers to efficiency of the universal and the taxon specific primers, which were obtained by standard curves; CT_univ_ and CT_spec_ are the CT values registered by the thermocycler (17).

### Quantification of bacteria and phage in feces.

We supplemented 0.2 g of fecal material with 1 mL of PBS which was mixed by vortex. The fecal solution was serially diluted into PBS, applied to a sorbitol MacConkey agar plate, and incubated at 37°C to count the colonies of STEC O157:H7 EDL933. To detect the phage loads in feces, the supernatant of the fecal fluid was filtered with a 0.22 μm sterile filter and analyzed by the plaque assay.

### Phage resistant test *in vivo*.

STEC O157:H7 EDL933 strain was isolated from the feces of infected mice treated with the phage cocktail (STEC + phage cocktail group) during days 5 to 7.The feces were diluted into PBS, applied to a sorbitol MacConkey agar plate to select STEC O157:H7 EDL933, which were confirmed by PCR using the primers listed in Table S1. Two-hundred colonies of STEC O157:H7 EDL933 were randomly selected for the phage resistance test by spot assay. STEC O157:H7 EDL933 were spread onto the LB agar plates, then 10 μL of phage cocktail solution (10^7^ PFU/mL) was spot on the bacterial lawns. After, the plates were incubated at 37°C for 12h. The absence of clear zoon was determined for phage resistance.

### Statistical analysis.

Statistical analyses were performed by using GraphPad Prism software (v 8.0). Experimental data were expressed as mean ± SD. The results for each experimental group were evaluated by multivariate one-way analysis of variance (ANOVA), followed by a Bonferroni’s test to compare the groups two by two. Survival curves were analyzed using the log-rank test. *P ≤ *0.05 was considered significant. Significance is indicated in the figures by asterisks (∗, *P < *0.05; ∗∗, *P < *0.01; ∗∗∗, *P < *0.001; ∗∗∗∗, *P < *0.0001).
