# Supplementary material for: Phage Cocktail Targeting STEC O157:H7 Has Comparable Efficacy and Superior Recovery Compared with Enrofloxacin in an Enteric Murine Model
Source: Microbiol Spectr. 2022 May 10;10(3):e00232-22. doi: 10.1128/spectrum.00232-22 (PMC9241756; doi:10.1128/spectrum.00232-22)
Supplement: SUPPLEMENTAL FILE 1 — Supplemental material. Download spectrum.00232-22-s001.pdf, PDF file, 0.6 MB [file spectrum.00232-22-s001.pdf]

Supplementary Materials

Journal name: Microbiology Spectrum

Phage cocktail targeting STEC O157:H7 has comparable efficacy and superior recovery compared to enrofloxacin in an enteric murine model

Yuxin Wang[1], Dinesh Subedi[2], Jin Li[1], Jiaoling Wu[1], Jianluan Ren[1], Feng Xue[1], Jianjun Dai[1,3], Jeremy J. Barr[2], Fang Tang[1]*

1. MOE Joint International Research Laboratory of Animal Health and Food Safety; Key Laboratory of Animal Bacteriology, Ministry of Agriculture; College of Veterinary Medicine, Nanjing Agricultural University, Nanjing, 210095, China
2. School of Biological Sciences, Monash University, 3800 Clayton, Victoria, Australia
3. School of Life Science and Technology, China Pharmaceutical University, Nanjing, China

Running Title: Comparison of phage and antibiotic treatment

*Corresponding author: Fang Tang

College of Veterinary Medicine, Nanjing Agricultural University, No.1 Weigang, Nanjing, 210095, Jiangsu province, P. R. China

Phone: +86(0) 25-84396517

Fax: +86(0) 25-84396517

E-mail: tfalice@126.com

MATERIALS AND METHODS

Morphology observation. Phage solutions were suspended in doubly deionized H_2O, transferred onto a copper grid, and subjected to negative staining with Phospho-tungstic acid (PTA, 2%, w/v). Phage morphology was observed using an H 7650 transmission electron microscope (TEM; Hitachi, Japan).

pH stability. One hundred microliter of phage cocktail was mixed with 900 μL of SM buffer in different pH values (2, 3, 4, 5, 6, 7, 8, 9, 10, 11, 12, 13, 14), then incubated in a water bathe at 37°C. Samples were taken out after 0.5 h and 1 h, and the potency was measured by double-layer agar method.

Thermal stability. One milliliter of phage cocktail was incubated in a water bath at 50°C, 60°C, and 70°C, respectively. Samples were taken out every 10 min to measure the titers by double-layer agar method.

Optimal multiplicity of infection (MOI). The host bacteria STEC O157:H7 EDL933 were cultured to the logarithmic phase and mixed with phage at different multiplicity of infection: 0.01, 0.1, 1, 10, and 100. After incubated at 37°C for 5h, the phage titer of supernatant was determined by double-layer agar method. The group with the highest titer is the MOI of the phage.

One-step growth curve. Host bacteria was cultured to the logarithmic phase (10^8 CFU/mL) and mixed with phage cocktail (10^7 PFU/mL) in equal volumes, incubated at 37°C for 15 min, centrifuged at 10,000rpm for 10 min. The pellet was resuspended in 5 mL of LB broth and incubated in a shaker at 37°C. For the first 15 min, 100 μL of sample was taken out every 5 min, and then every 15 min until 120 min. Samples were centrifuged at 10,000 rpm for 1 min, and the supernatants were diluted into an appropriate gradient to test the titer by double-layer agar method.

Table S1 Primers for detection of STEC O157:H7 (1, 2).

Primers	Primer sequences (5'-3')	Target gene	Annealing temperature (℃)	Product size (bp)
O157-F	AAGATTGCGCTGAAGCCTTTG	*rfbE*	55	497
O157-R	CATTGGCATCGTGTGGACAG			
H7-F	GCGCTGTCGAGTTCTATCGAGC	*fliC*	55	625
H7-R	CAACTGTGACTTTATCGCCATTCC			

Table S2 16S rRNA gene-targeted group-specific primers used in this study (3).

Primers	Primer sequences (5'-3')	Target group	Product size (bp)
Bac-F	GTTTAATTCGATGATACGCGAG	*Bacteroidetes*	122
Bac-R	TTAASCCGACACCTCACGG		
Firm-F	GGAGYATGTGGTTTAATTCGAAGCA	*Firmicutes*	126
Firm-R	AGCTGACGACAACCATGCAC		
Act-F	TGTAGCGGTGGAATGCGC	*Actinobacteria*	277
Act-R	AATTAAGCCACATGCTCCGCT		
Sac-F	AAGAGAACTGTGCCTTCGG	"*Candidatus Saccharibacteria*"	187
Sac-R	GCGTAAGGGAAATACTGACC		
Defer-F	CTATTTCCAGTTGCTAACGG	*Deferribacteres*	150
Defer-R	GAGHTGCTTCCCTCTGATTATG		
Ver-F	TCAKGTCAGTATGGCCCTTAT	*Verrucomicrobia*	97
Ver-R	CAGTTTTYAGGATTTCCTCCGCC		
Ten-F	ATGTGTAGCGGTAAAATGCGTAA	*Tenericutes*	200
Ten-R	CMTACTTGCGTACGTACTACT		
Beta-F	AACGCGAAAAACCTTACCTACC	*Betaproteobacteria*	174
Beta-R	TGCCCTTTCGTAGCAACTAGTG		
Epsilon-F	TAGGCTTGACATTGATAGAATC	*Epsilonproteobacteria*	189
Epsilon-R	CTTACGAAGGCAGTCTCCTTA		
Gamma-F	GCTAACGCATTAAGTRYCCCG	*Delta- and Gammaproteobacteria*	189
Gamma-R	GCCATGCRGCACCTGTCT		
Uni-F	AAACTCAAAKGAATTGACGG	Universal	136
Uni-R	CTCACRRCACGAGCTGAC		

Note: Nucleotide symbols: R = A or G; Y = C or T; N = any nucleotide; W = A or T; M = A or C; K = T or G; S = C or G; and H = A/C/T.

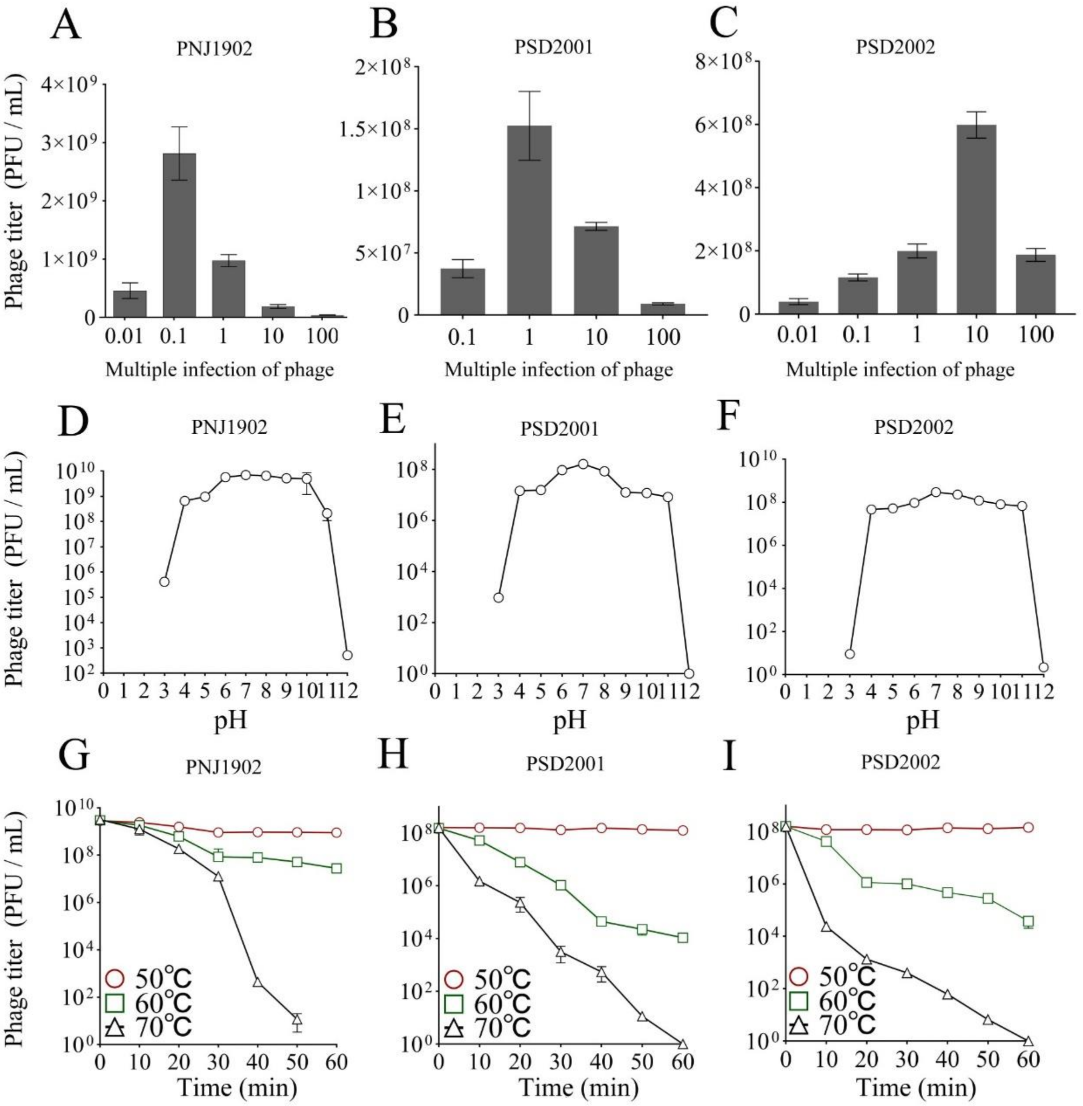

Fig.S1 Optimal MOI, pH and temperature resistance of phages. Optimal MOI of the phage PNJ1902**(A)**, PSD2001**(B)**, PSD2002**(C)**; pH stability of the phage PNJ1902**(D)**, PSD2001**(E)**, PSD2002**(F)**; Temperature tolerance of phage PNJ1902**(G)**, PSD2001**(H)**, PSD2002**(I)**.

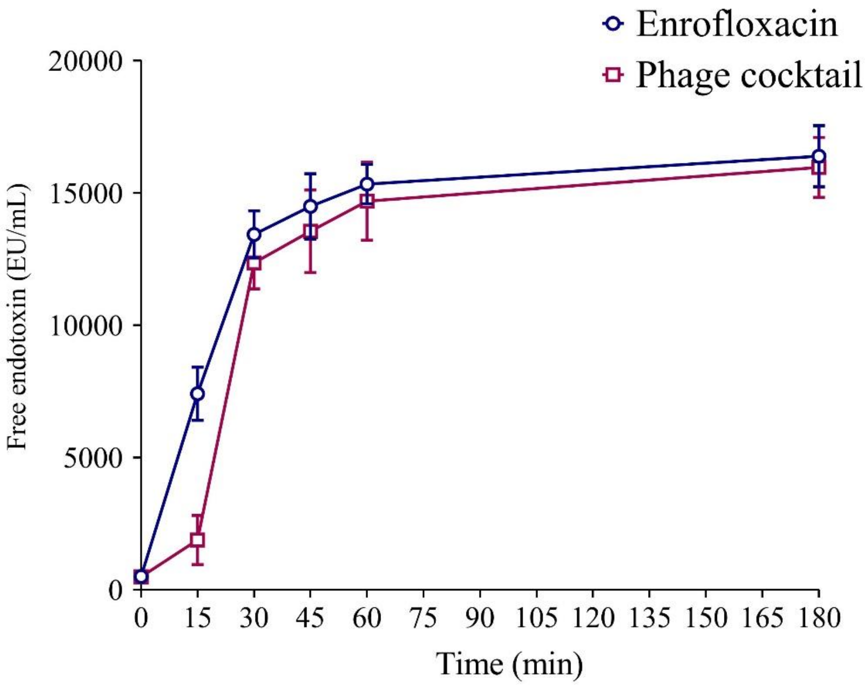

Fig. S2 Concentration of released endotoxin**.** The concentration of endotoxin released by STEC exposed to enrofloxacin, and phage cocktail *in vitro* was tested. The data is represented by mean ± SD.

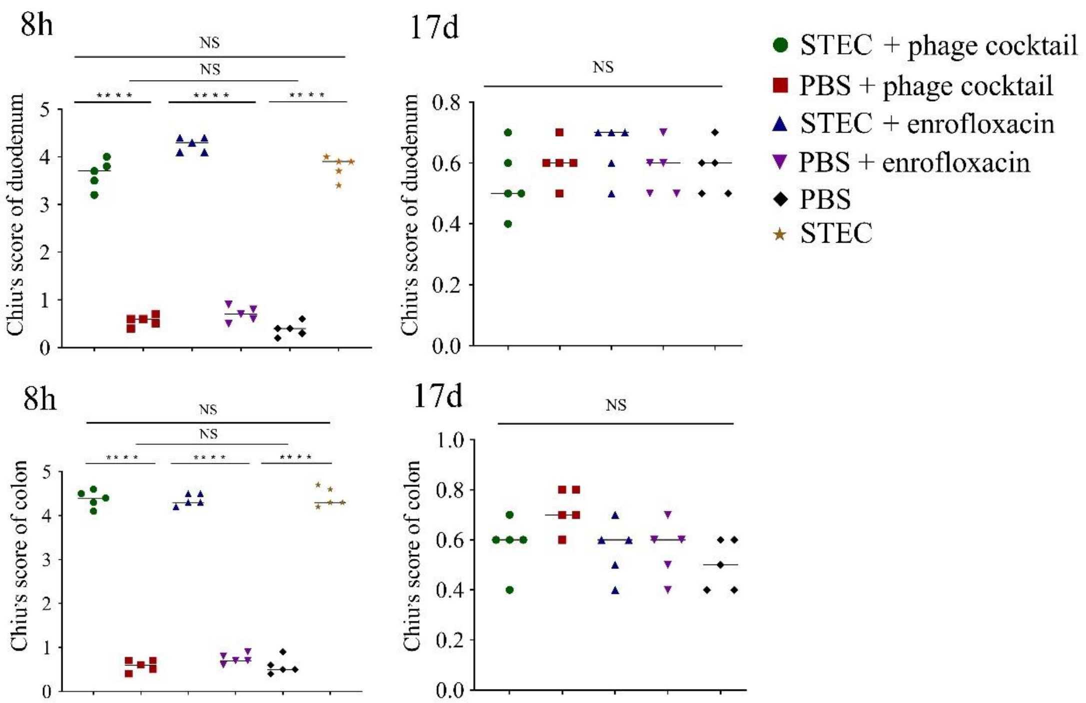

Fig. S3 Chiu's analysis of mouse gut at 8h and 17d. Chiu's Pathology score was used to quantify the pathological changes of duodenum and colon of mice. Statistical analyses were performed by one-way analysis of variance (ANOVA). Significant difference between STEC+ enrofloxacin and STEC+ phage cocktail is indicated in the figures by asterisks (*, $P < 0.05$; **, $P < 0.01$; ***, $P < 0.001$; ****, $P < 0.0001$).

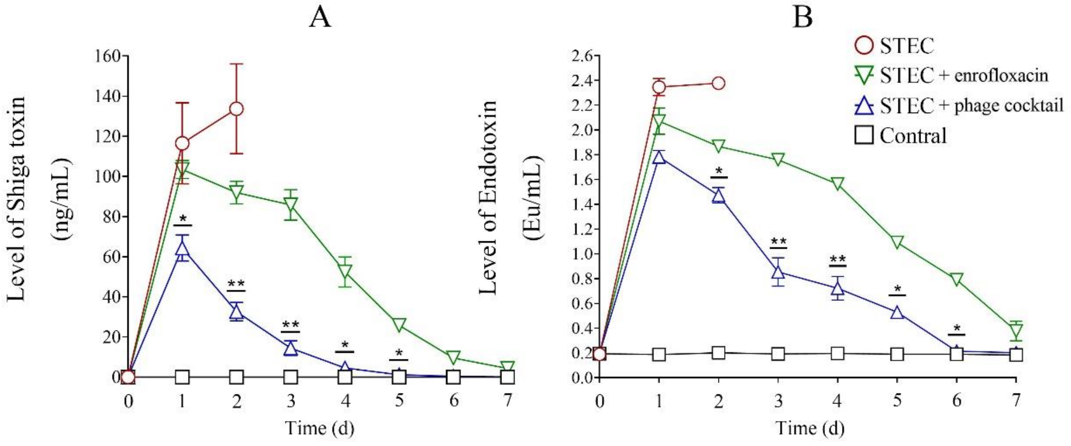

Fig.S4 Shiga toxin and endotoxin released in mice serum. Serum of mice in different groups, including STEC+ enrofloxacin, STEC+ phage cocktail, STEC and blank control were collected, and the concentration of Shiga toxin **(A)** and endotoxin **(B)** were detected. Statistical analyses were performed by one-way analysis of variance (ANOVA). Significant difference between STEC+ enrofloxacin and STEC+ phage cocktail is indicated in the figures by asterisks (∗, $P < 0.05$; ∗∗, $P < 0.01$).

References

1. García-Sánchez, A., Sánchez, S., Rubio, R., Pereira, G., Alonso, J. M., Mendoza, J. , Rey, J. 2007. Presence of Shiga toxin-producing *E. coli* O157:H7 in a survey of wild artiodactyls. Vet Microbiol 121, (3-4): 373-377. https://doi.org/10.1016/j.vetmic.2006.12.012
2. Sánchez, S., Martínez, R., García, A., Blanco, J., Echeita, A., Mendoza, J., Rey, J. , Alonso, J. M. 2010. Shiga toxin-producing *Escherichia coli* O157:H7 from extensive cattle of the fighting bulls breed. Res Vet Sci 88(2): 208-210. https://doi.org/10.1016/j.rvsc.2009.07.014
3. Yang YW, Chen MK, Yang BY, Huang XJ, Zhang XR, He LQ, Zhang J, Hua ZC. 2015. Use of 16S rRNA gene-targeted group-specific primers for real-time PCR

analysis of predominant bacteria in mouse feces. Appl Environ Microbiol 81:6749 – 6756. https://doi.org/10.1128/AEM.01906-15.
